# Ex Vivo and in Vivo Study of Sucrosomial^®^ Iron Intestinal Absorption and Bioavailability

**DOI:** 10.3390/ijms19092722

**Published:** 2018-09-12

**Authors:** Angela Fabiano, Elisa Brilli, Letizia Mattii, Lara Testai, Stefania Moscato, Valentina Citi, Germano Tarantino, Ylenia Zambito

**Affiliations:** 1 Department of Pharmacy, University of Pisa, 56126 Pisa, Italy; angyfab@gmail.com (A.F.); lara.testai@unipi.it (L.T.); valentina.citi@unipi.it (V.C.); ylenia.zambito@unipi.it (Y.Z.); 2 Pharmanutra S.p.A., 56122 Pisa, Italy; e.brilli@pharmanutra.it (E.B.); g.tarantino@pharmanutra.it (G.T.); 3 Department of Clinical and Experimental Medicine, University of Pisa, 56126 Pisa, Italy; stefania.moscato@unipi.it; 4 Interdepartmental Research Center Nutraceuticals and Food for Health, University of Pisa, 56124 Pisa, Italy

**Keywords:** iron bioavailability, self-assembled vesicles, sucrester, lecithin, iron storage

## Abstract

The present study aimed to demonstrate that Sideral^®^ RM (SRM, Sucrosomial^®^ Raw Material Iron) is transported across the excised intestine via a biological mechanism, and to investigate the effect that this transport route may produce on oral iron absorption, which is expected to reduce the gastrointestinal (GI) side effects caused by the bioavailability of non-absorbed iron. Excised rat intestine was exposed to fluorescein isothiocyanate (FITC)-labeled SRM in Ussing chambers followed by confocal laser scanning microscopy to look for the presence of fluorescein-tagged vesicles of the FITC-labeled SRM. To identify FITC-labeled SRM internalizing cells, an immunofluorescence analysis for macrophages and M cells was performed using specific antibodies. Microscopy analysis revealed the presence of fluorescein positive particulate structures in tissues treated with FITC-labeled SRM. These structures do not disintegrate during transit, and concentrate in macrophage cells. Iron bioavailability was assessed by determining the time-course of Fe^3+^ plasma levels. As references, iron contents in liver, spleen, and bone marrow were determined in healthy rats treated by gavage with SRM or ferric pyrophosphate salt (FP). SRM significantly increased both area under the curve (AUC) and clearance maxima (C_max_) compared to FP, thus increasing iron bioavailability (AUC_rel_ = 1.8). This led to increased iron availability in the bone marrow at 5 h after single dose gavage.

## 1. Introduction

Iron deficiency is one of the most widespread nutritional deficiencies [1]. Oral supplementation of iron deficiency is mainly based on ferrous iron formulations [2]. However, numerous gastrointestinal side effects, including an increased risk of intestinal inflammation, constipation, and diarrhea, consequently affecting the microbiota, have been reported [3,4,5,6,7,8]. Generally, iron supplements are, for the most part, unabsorbed in the duodenum and this results in poor iron bioavailability. In fact, too much unabsorbed iron may stimulate the virulence of pathogens present in the intestine, and contribute to the creation of a pro-inflammatory oxidative environment [9]. Despite its crucial role in cellular processes, the presence of free iron can generate toxic free radicals and oxygen reactive species, which can impair the integrity of intestinal epithelium by promoting redox stress [10]. Such a compromised integrity was indicated by in vitro studies on Caco-2 cells exposed to iron [11,12]. To enhance the absorption of administered iron, and at the same time ruling out gastrointestinal (GI) side effects, it could be very useful to develop an oral formulation able to carry the ferric ion from the administration site down to the intestine. There, after crossing the intestinal epithelium, the iron should reach the bloodstream [13]. Recently our group has shown that Sideral^®^ RM (SRM, Sucrosomial^®^ Raw Material Iron) is able to (1) retain the iron within the Sucrosome in the acidic environment of the stomach, (2) promote iron absorption by Caco-2 cells, (3) protect Fe^3+^ from reduction by intestinal enzymes, and (4) promote Fe^3+^ transport across the intestinal epithelium unmediated by the divalent metal transporter 1 (DMT-1) carrier [14]. In that study, evidence was produced strongly suggesting that the two iron forms, Fe^2+^ and Fe^3+^, follow two distinct absorption routes; namely, Fe^2+^ transport is mainly carrier-mediated, whereas Fe^3+^ from SRM is partly encapsulated in particulate structures able to penetrate the intestinal barrier by a biological mechanism, which is a different transport route [14]. In fact, the ability of SRM to promote Fe permeation across the excised rat intestine was shown in vitro and ex vivo [14]. Yet, although an ex tempore formation of vesicles was argued, neither the fate of the particle in the course of permeation across the intestinal tissue nor the possible impact of particle absorption on oral iron bioavailability was made clear. In light of the results obtained in the previous study, the purposes of the present one have been the following: (1) to confirm the SRM product actually undergoes transcytosis across the excised intestine barrier, (2) to investigate the effect the transport through the intestinal tissue may have on the SRM particles and (3) to investigate the improvement in oral iron absorption that may be produced by this transport route, which is expected to reduce the GI side effects caused by non-absorbed iron, and increase the iron bioavailability. To this purpose SRM was studied on a bioavailability basis using an in vivo rat model.

## 2. Results and Discussion

### 2.1. Study of Fluorescein Isothiocyanate (FITC)-Labeled SRM Internalization in and across Intestinal Barrier

**Figure 1:**
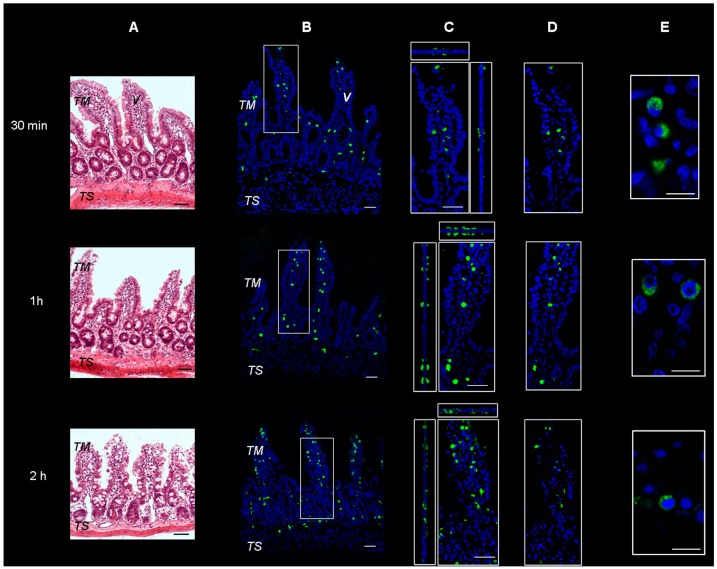
Representative images of rat gut sections after 0.5 h, 1 h and 2 h incubation in Ussing chamber. TM: tunica mucosa; TS: tunica serosa; V: intestinal villus. (**A**) Hematoxylin and eosin stain. Scale bars 50 µm. (**B**–**E**) Immunofluorescence confocal microscopy; individual fluorescent channels: green (SRM, Sucrosomial^®^ Raw Material Iron) and blue (nuclei). (**B**) Bidimensional image of the maximum intensity projection. Scale bar 50 µm. (**C**) Blow-up of the squares shows higher magnification of the three-dimensional pictures with the respective orthogonal projections. Scale bar 50 µm. (**D**) Images acquired in one Z-plane (31st of 48, 46th of 75, and 33rd of 65, incubated for 0.5, 1 and 2 h respectively). Scale bar 50 µm. (**E**) Images acquired in one Z-plane (28th of 40, 15th of 46, 28th of 47, incubated for 0.5, 1 and 2 h, respectively) at a magnification of different sections higher than that of (**B**–**D**). Scale bar 10 µm.

Microscopical observation on hematoxylin and eosin-stained sections of rat intestine revealed a conserved normal morphology of the tissues, as can be observed in Figure 1A. Sections of intestinal wall were also observed on a confocal microscope following incubation with FITC-labeled SRM. The representative images, seen in Figure 1B–D, show green fluorescent spots spanning the whole tissue thickness after all incubation times tested. This is indicative of particulate structures generated by the samples and penetrating into the tissue from the mucosal to serosal side, not disintegrating during transit. Due to the supramolecular nature of the penetrant, the transepithelial penetration mechanism is believed to be of the biological, not the physico-chemical type.

The results obtained by measuring the fluorescent mass fraction in an Ussing chamber, demonstrated that the fluorescence was going through the excised rat intestinal wall, as the fluorescence significantly increased in the receiving compartment with increasing incubation times (0.5, 1 and 2 h of incubation, i.e., 0.8 ± 0.1, 1.0 ± 0.2 and 4.9 ± 0.3% respectively). This fluorescence increase was in agreement with data on Fe^3+^ permeation across and retention in excised intestinal tissue already reported and discussed in our previous study [14].

**Figure 2:**
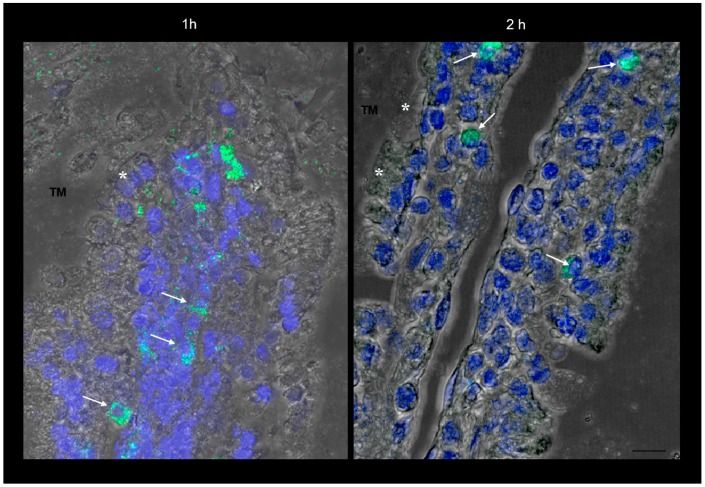
Confocal laser scanning microscopy: representative images of rat gut sections after 1 and 2 h incubation in an Ussing chamber. Asterisks indicate enterocytes; arrows indicate connective cells. TM: tunica mucosa. Individual fluorescent channels: green (SRM) and blue (nuclei). Scale bar 10 µm.

The more magnified images in Figure 1E and Figure 2 show that the green fluorescence is due to particulate structures. These are observed in enterocytes and in connective tissue cells incubated with SRM up to 1 h, while after 2 h incubation, fluorescent particles are only localized in connective cells. This is a clear sign of particle transit across the full thickness of the intestinal barrier, which would mainly be governed by a biological mechanism.

**Figure 3:**
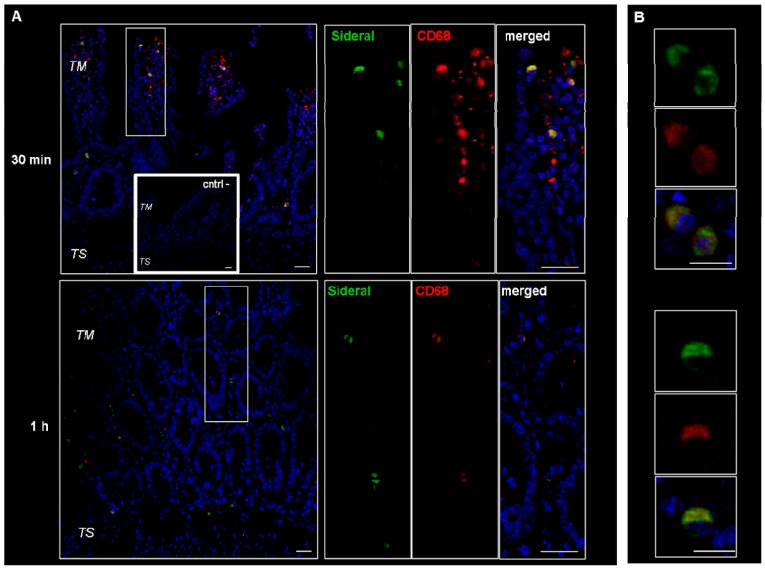
Confocal laser scanning microscopy: representative images of rat gut sections after 0.5 and 1 h incubation in Ussing chamber. Negative controls obtained omitting primary antibodies are shown in double line squares. Individual fluorescent channels: green (SRM), red (CD68 + cells) and blue (nuclei). (**A**) Bidimensional image of the maximum intensity projection. Scale bar 25 µm. (**B**) Images acquired in one Z-plane (29th of 47 and 26th of 40, incubated 0.5 and 1 h, respectively) at a magnification of different sections higher than that of (A). Scale bar 10 µm.

**Figure 4:**
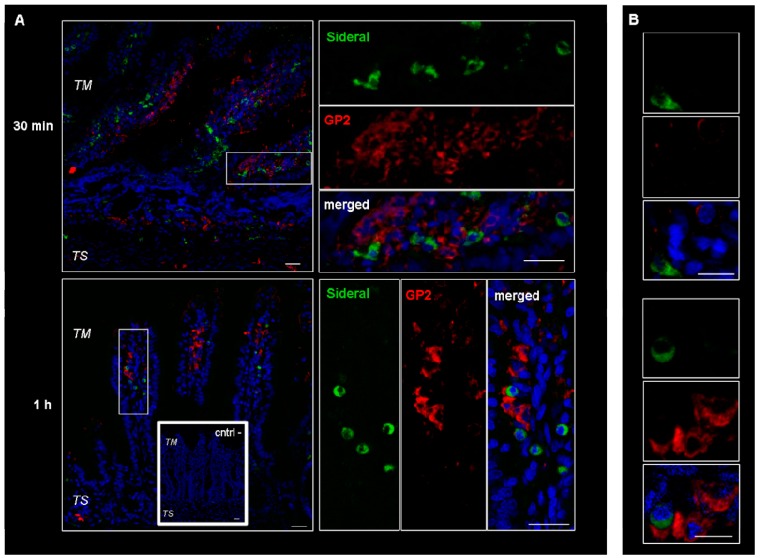
Confocal laser scanning microscopy: representative images of rat gut sections after 0.5 and 1 h incubation in Ussing chamber. Negative controls obtained omitting primary antibodies are shown in double line squares. Individual fluorescent channels: green (SRM), red (GP2 + cells) and blue (nuclei). (**A**) bidimensional image of the maximum intensity projection. Scale bar 25 µm. (**B**) Images acquired in one Z-plane (38th of 59, 22nd of 64, incubated 0.5 h and 1 h, respectively) at a higher magnification. Scale bar 10 µm.

These cells were supposed to be macrophages and M cells. However, since macrophages change their shape from an elongate-fusiform to star-shaped, the morphology of these cells does not per se allow their clear identification. Therefore, immunonofluorescence analysis was carried out to assess the expression of such antigens as CD68 and Gp2 specific to macrophages and M cells respectively. As evidenced by the representative examples in Figure 3, the particulate structures (green) are concentrated in CD68 positive (red) cells. Indeed, in the merged images obtained by superimposing the two channels (green and red), the system-containing cells appear yellow due to such superimposition. Consequently, CD68 positive cells appear to be involved in the internalization process. This finding is in agreement with our previous study [14], where it was observed that the amount of Fe^3+^ accumulating in intestinal tissue was higher than the cumulative amount passing into the receiving phase of the Ussing chamber. Such an accumulation is probably due to the particle internalization by macrophages observed in Figure 3. On the other hand, as shown in Figure 4, this is not the case with the GP2 positive cells, at least within the limits of the incubation terms tested. Macrophages are supposed to store SRM, as they primarily do with erythrophagocytosis-acquired iron, subsequently releasing it into the plasma when needed. Therefore, the significance of macrophages in SRM transport could be as transient iron storage.

### 2.2. Bioavailability Studies

In the preceding sections, evidence was given for the biological process of the SRM-carried Fe^3+^ iron penetration across excised intestine epithelium, which had already been postulated in a previous study by our group [14]. The purpose of the present in vivo experiments has been to assess the impact this additional absorption route may have on oral iron bioavailability. Healthy rats were used, although evidencing differences in the profiles for the different samples tested was expected to be much more difficult because of the natural iron homeostasis in healthy animals [15]. In view of the possible side effects of macrophage activation upon rat exposition to SRM, the main vital functions were continuously monitored by electrocardiogram. This allowed the ruling out of any severe inflammatory reactions. Nevertheless, further in vivo studies will have to be performed on SRM-treated rats to better clarify this point.

**Figure 5:**
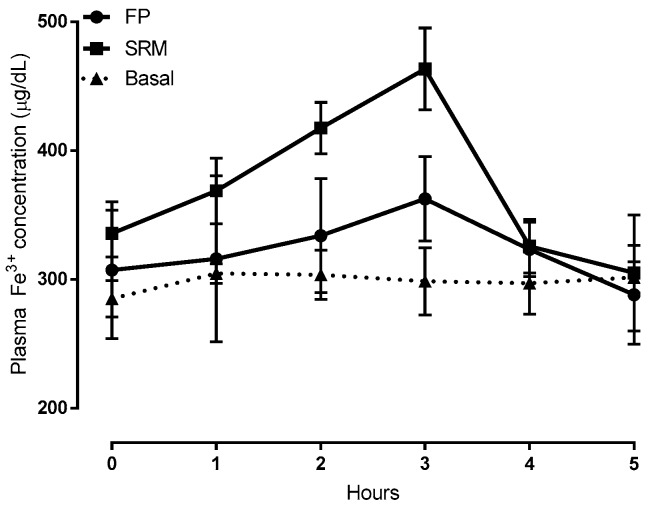
Plasma Fe^3+^ concentration vs time plots following administration of 400 μL of a suspension (5 mg/kg of iron) of SRM and ferric pyrophosphate salt (FP, ferric pyrophosphate salt) (control) compared to Fe^3+^ concentration in untreated animals (basal). Means ± SD of at least six values obtained with different animals.

Time-courses of plasma iron concentration up to 5 h after the oral administration of formulations SRM and ferric pyrophosphate salt (FP) compared to those of untreated rats, are depicted in Figure 5. Compared to FP, pharmacokinetic profiles showed that the SRM formulation could significantly increase both area under the curve (AUC) and clearance maxima (C_max_), thus increasing iron bioavailability, as shown by data in Table 1. These data are the means of at least six animals.

Considering the results from our previous work [14] and those obtained so far in the present one, it can be stated that the SRM formulation can increase the apparent permeability of Fe^3+^ across the excised rat intestinal epithelium through the formation of particles able to penetrate across such a barrier without disintegrating, thus adding a new iron trans-epithelial penetration route to those already known and increasing oral iron bioavailability. The data also suggest that the particles evidenced in Figure 1 and Figure 2 contained Fe^3+^.

**Figure 6:**
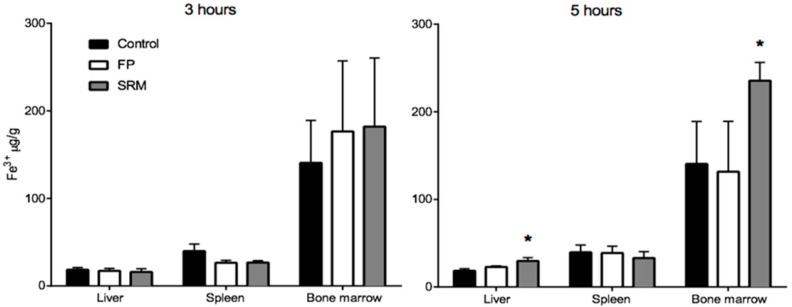
Quantity (μg) of Fe^3+^ per gram of organ withdrawn from rats sacrificed after 3 or 5 h from the gavage. * *p* < 005. Means ± SD of at least six values obtained with different animals.

Figure 6 shows the Fe^3+^ content in different tissues from rats treated with FP, SRM and saline 3 h and 5 h after administration. Tissues from animals treated with FP do not show significant increase in Fe^3+^ content over the control, whereas a significant increase in Fe^3+^ content is observed in the liver and in the bone marrow of animals treated with SRM 5 h after administration. The relevant enhancement factor (EF), calculated as the ratio between the amount of Fe^3+^ found in the liver and bone marrow after SRM administration and the corresponding amount found in non-treated rats (control), is 1.6 and 1.7, respectively. This data points to an Fe^3+^ accumulation in liver and bone marrow consequent to SRM administration, meaning that the iron exceeding the requirements of the organs and various metabolic processes is stored in the hepatocytes [16]. Moreover, the blood samples themselves that were withdrawn during in vivo studies, i.e., a total of 3.5 mL (30–35% of total blood in a rat) might have stimulated the production of erythrocytes by the bone marrow. This could be an additional cause of Fe^3+^ accumulation in this tissue. According to a literature report, most of the iron is in fact transported to the bone marrow to produce erythrocytes; a smaller portion reaches other tissues for other fundamental cellular processes, and the excess is stored in the liver in the form of ferritin iron [17]. However, in no case was a significant increase in Fe^3+^ mass in liver and bone marrow observed 3 h after iron administration. This can be explained by considering that the liver, in addition to acting as a reservoir of excess iron, also acts as the main iron homeostasis control [16]. Thus, only after iron has attained its peak plasma concentration will it start accumulating in the liver. Moreover, Pantopoulos et al. (2012) reported that macrophages, which were seen in Figure 3 to be involved in internalization of Fe^3+^-loaded particles in intestinal tissue, are a further site of iron storage in the organism [18]. 

Hence, it can be stated that SRM is able not only to increase iron bioavailability, but also to keep correct levels of Fe^3+^ in the blood for longer than FP.

**Figure 7:**
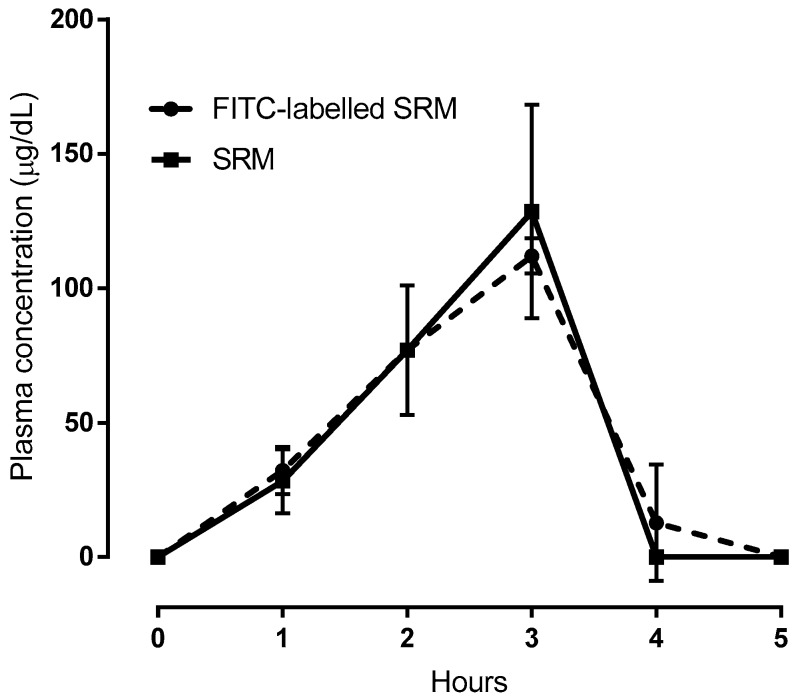
Comparison between plasma concentration vs time profiles following administration of SRM or FITC-labeled SRM. In case of SRM plasma was analyzed for Fe^3+^; in case of FITC-labeled SRM plasma was analyzed for FITC. Means ± SD of three values obtained with different animals.

In vivo bioavailability studies were also carried out on FITC-labeled SRM samples. To this end, one of the SRM excipients that had been suggested to be responsible for vesicle formation was monitored after labeling with FITC. In fact, the data represented in Figure 7, net of the basal Fe^3+^ concentration values, highlight the virtual superimposition of the plasma concentration versus time profile determined for the FITC-labeled excipient and the Fe^3+^. Since the iron and labeled excipient contents formulated in SRM are equal, the data superimposition in Figure 7 could well be explained by admitting that a fraction of the administered iron-loaded SRM formulation traveled unmodified from its application site along the GI tract and across the intestinal barrier to reach the bloodstream. Quantifying such a fraction, however, was deemed to be of modest interest for the present study, as the in vivo experiments were carried out with animals, not humans. Therefore, the ex tempore formation of nano-vesicles upon SRM contact with physiological fluids and vesicle intestinal absorption by a biological mechanism, not accompanied by any vesicle impairment, is a faithful interpretation of data.

## 3. Materials and Methods 

### 3.1. Materials

Pepsin, pancreatin, Chelex-100 resin, fluorescein isothiocyanate (FITC), hematoxylin and eosin were all from Sigma–Aldrich, while polyester membrane filters (pore size 0.4 μm, area 1.12 cm^2^) were from Celbio, Milano, Italy. Sucrester was from Chimab S.p.A., Italy, and thiopental was from MSD (Animal Health, Milan, Italy). The carbon dioxide/oxygen (95/5 *v*/*v*) mixture (Oxycarb) was from Sol, Pisa, Italy. Mouse anti-CD68 monoclonal antibody (ab201340), used to identify macrophages, was from Abcam (Cambridge, UK). Rabbit anti-GP2 polyclonal antibody (PA5-42593), used to identify M cells, was from ThermoFisher scientific (Waltham, MA, USA). Alexa Fluor^®^ 568 donkey anti-mouse IgG, Alexa Fluor^®^ 568 donkey anti-rabbit IgG secondary antibodies and Nuclear dye TO-PRO (TO-PRO^®^-3 stain) were purchased from Life Technologies Italia (Monza, Italy). Sideral^®^ RM (Sucrosomial^®^ Iron, SRM), purchased from Alesco s.r.l., is now present on the market. According to the manufacturer, Sideral^®^ RM is a source of ferric pyrophosphate covered by a matrix of phospholipids and sucrose esters of fatty acids. Ferric pyrophosphate salt (FP) was used as a reference. 

None of the powders tested contained any particles so small as to pass through a 106 µm sieve. All chemicals and solvents used in this work were of reagent grade.

### 3.2. Fluorescein Isothiocyanate Labeling of Sucrester

A previously described procedure was followed [19]. A solution of FITC in dimethyl sulfoxide (0.2 mL, 2 mg/mL) was added to an aqueous solution of sucrester (20 mL, 2 mg/mL), and the mixture incubated for 8 h at 4 °C. The solution was then passed through a column of Sephadex G15 to clear the labeled polymer of nonreacted FITC, and lyophilized. In no case did the Sephadex column retain any fluorescence, indicating the absence of nonreacted FITC in all cases and the complete labeling of sucrester. Hence, the fluorophore bound to the sucrester could be calculated at 1% of the total mass. This sucrester was used to prepare FITC-labeled SRM.

### 3.3. Biopharmaceutical Procedures

All the experimental procedures were carried out following the guidelines of the European Community Council Law 2010/63, the Italian law (D.L. 2014/26), and were approved by the Ministry of Health (n°670/2016-PR), Rome, Italy. All procedures on rats were approved by the Italian Ministry of Health, approval date 7th July, 2016 (number 670/2016-PR). 

#### 3.3.1. Study of FITC-Labeled SRM Internalization in and across Intestinal Barrier

A previously described procedure was followed [20]. Briefly, the intestine of non-fasting male Wistar rats was used. After sacrificing the rats, the first 20 cm of jejunum was immediately removed. The excised intestine was mounted in Ussing type chambers (0.78 cm^2^ exposed surface area) and after 20 min equilibration, the medium in the donor compartment was replaced by 1 mL of pre-equilibrated 64.2 mg/mL FITC-labeled SRM dispersion in phosphate buffered saline (PBS) pH 7.4, 0.13 M, containing 8.4 mg of iron in the form of ferric pyrophosphate salt. This dispersion was prepared by shaking the appropriate sample amount in simulated gastric digestion fluids to simulate sample transit across the stomach as previously described [21]. After a pre-determined term, the receiving phase was analyzed for fluorescence (Perkin Elmer L50 spectrofluorometer, 490 nm excitation, 520 nm emission wavelengths). To observe the incubated intestinal tissue under a fluorescence microscope after 0.5, 1, or 2 h, incubation strips of intestinal tissue were fixed in 4% phosphate-buffered paraformaldehyde overnight at 4 °C. Samples were then dehydrated and embedded in paraffin according to standard histological techniques. Sections 8 μm thick were cut across the long axis of the gut lumen to obtain sections of whole thickness of the intestinal wall, from the mucosal to serosal layer. Sections mounted on glass slides were deparaffined, rehydrated and incubated for 15 min with nuclear dye TO-PRO (1:1000 in PBS). After sealing with PBS/glycerol solution (1:2), the slides were examined by means of a confocal laser scanning microscope (TC SSP8 Leica Microsystems, Mannheim, Germany) using ×20, ×40 and ×63 oil immersion lenses and 488 nm and 642 nm excitation wavelength lasers. The analysis of the fluorescence pattern was performed in at least three non-consecutive sections for each sample. In addition, hematoxylin and eosin staining was used to verify the integrity of the tissues. In order to identify SRM receiving cells, immunofluorescence analysis for macrophages and M cells was performed on strips after 0.5 or 1 h incubation in an Ussing chamber. In brief, slides with de-paraffined sections were incubated in blocking solution (0.1% Tween, 0.25% BSA in PBS) for 1 h, and then with primary antibodies diluted in blocking solution 1:200 (anti-CD68) or 1:120 (anti-GP2) overnight at 4 °C. After three washes, slides were incubated 1.5 h in the dark with relative fluorescent secondary antibodies diluted 1:250 in blocking solution. Nuclear staining was performed by TO-PRO. Samples were mounted with PBS-glycerol solution. Negative controls for secondary antibodies were performed omitting primary antibodies and incubating the specimens with nonimmune serum. Sections were observed under confocal laser scanning microscopy using ×20, ×40, and ×63 oil immersion lenses and 488 nm, 561 nm and 642 nm excitation wavelength lasers.

#### 3.3.2. Bioavailability Studies

The time-course of the plasma levels of Fe^3+^ was evaluated using male Wistar rats (260–350 g), anesthetized with sodium thiopental (70 mg/kg, i.p.). Artificial ventilation with room air (stroke volume 1 mL/100 g body weight; 70 strokes/min) was maintained by a rodent ventilator (mod 7025 Ugo Basile, Comerio, Italy) after trachea intubation. The main vital functions were continuously monitored by lead II of an electrocardiogram (ECG) (Mindray, PM5000; 2 Biological Instruments, Varese, Italy). Blood samples (500 μL) were collected (basal control) after a 15 min stabilization time. Then, at 30 min and 1, 2, 3, 4 and 5 h after gavage (about 400 μL corresponding to 5 mg/kg of iron-containing SRM, FITC-labeled SRM or FP), samples were analyzed for Fe^3+^ or fluorescence. Each blood sample was immediately centrifuged (2040× *g*, 20 min, 4 °C) and the serum separated (150 μL) and analyzed for Fe^3+^, as previously described [22].

In case of FITC-labeled SRM, the serum was analyzed using the fluorimeter (Perkin Elmer LS 45) at λ_ecc_ 490 nm e λ_em_ 520 nm. For comparison, the Fe^3+^ concentration in the serum of three untreated animals was monitored (basal control). 

The area under the curve (AUC) of Fe^3+^ or FITC-labeled SRM concentration in the blood versus time and over the level of 238 µg/dL, which corresponds to the mean basal Fe^3+^ concentration found in rats at time 0 before administration of the formulations under study, was calculated by the linear trapezoidal rule between time 0 and 5 h. After completion of the bioavailability study, animals were sacrificed and liver, spleen and bone marrow were withdrawn and homogenized for 10 min at 8000 rpm with a PBS made to pH 3 [23]. The homogenate was centrifuged for 5 min at 10,000 rpm and the supernatant analyzed for Fe^3+^ content [22].

For reference the above procedure was repeated six times on liver, spleen, and bone marrow of control rats.

### 3.4. Statistical Data Treatment

Experiments were replicated (*n* = 3–6), the results were averaged and the statistical significance of differences between means was assessed by the Student’s *t*-test. Differences were considered significant, i.e., the null hypothesis was rejected, for p values lower than 0.05.

## 4. Conclusions

Ex vivo experiments showed that when SRM comes into contact with aqueous fluids, it extemporaneously forms vesicular-like structures that are internalized by epithelial cells. The studies with the confocal laser scanning microscope clarified that these vesicles remain intact during transit through the entire tissue, while the immunofluorescence studies showed that these vesicles, once internalized by epithelial cells, are concentrated in macrophages.

Although the in vivo experiments were conducted on healthy rats where normal iron homeostasis is guaranteed, the SRM formulation has shown an ability to significantly increase sideremia compared to FP. In agreement with a previous study by the present authors, this is ascribed to a promotion of higher intestinal iron absorption by SRM. This bioavailability enhancement leads to increased iron concentration in the liver and in the bone marrow. In this work we have proved that the increase in iron bioavailability is due to the extemporaneous formation in the GI tract of iron-loaded (Sucrosome) vesicles that can pass intact through the intestinal tissue. Moreover, we demonstrated that macrophages could be an adjunctive site of SRM storage. Although we did not observe any evident acute inflammatory reactions in SRM-treated rats, further studies are in progress to better understand whether the possible macrophage activation after GI exposure to SRM may lead to side effects. 

## Figures and Tables

**Table 1:** Pharmacokinetic data in the blood following administration of 400 µL of a suspension (5 mg/kg of iron) of SRM and FP.

Item	C_max_ (μg/mL)	t_max_, h	AUC_0–5_ (μg h/mL)	AUC_rel_
FP	352.7 ± 32.1	3	388.3 ± 151.6	-
SRM	463.5 * ± 31.8	3	694.5 * ± 57.2	1.8

* *p* < 0.05.
